# Hypercalcemia-Induced ST-Segment Elevation Mimicking Acute Myocardial Injury: A Case Report and Review of the Literature

**DOI:** 10.1155/2020/4159526

**Published:** 2020-03-16

**Authors:** Ashraf Abugroun, Aneesh Tyle, Farah Faizan, Michael Accavitti, Chaudhary Ahmed, Theodore Wang

**Affiliations:** ^1^Wayne State University, Detroit, Michigan, USA; ^2^Advocate Illinois Masonic Medical Center, Chicago, IL, USA; ^3^Jinnah Sindh Medical University, Karachi, Pakistan

## Abstract

ST-segment elevation in absence of acute coronary syndrome can be seen in multiple conditions, including acute pericarditis and coronary vasospasm, but it is rarely seen with severe hypercalcemia. The authors present a case of an 81-year-old female with a history of stage 4 squamous cell cancer of the lung, who presented to the emergency room with profound fatigue, weakness, anorexia, and drowsiness two weeks after her first chemotherapy cycle. Additionally, she had complaints of right-sided chest pain associated with worsening shortness of breath, as well as right arm numbness. An EKG obtained on arrival to the hospital showed diffuse ST-segment elevation (leads V3–V6, I, II, III, and aVF). Basic lab work found a calcium level of 20.4 mg/dl with elevated parathyroid hormone-related protein (PTHrP) of 135 pg/ml. Troponin I remained within normal limits. Serial EKS obtained during the patient's hospitalization demonstrated resolution of the ST elevation as calcium level normalized. This case emphasizes the importance of hypercalcemia as a differential diagnosis for ST-segment elevation and QT shortening when acute coronary syndrome is not present. Awareness of these EKG changes is critical for early diagnosis, recognition, and appropriate treatment.

## 1. Introduction

ST-segment elevation in the absence of acute coronary syndrome can be seen in multiple conditions including acute pericarditis, coronary vasospasm, early repolarization, and increased intracranial pressure [1]. In rare instances, ST-segment elevation has been recognized in the setting of severe hypercalcemia. Previous studies, mostly limited to case reports and case series, have demonstrated such an association. We herein report a case of diffuse ST-segment elevation mimicking acute coronary syndrome in the setting of severe hypercalcemia caused by metastatic squamous cell carcinoma. Serial EKGs demonstrated resolution of the ST changes concurrent with normalization of the serum calcium level. This case emphasizes the importance of recognition of hypercalcemia as an important cause for ST-segment elevation.

## 2. Case Report

**Figure 1:**
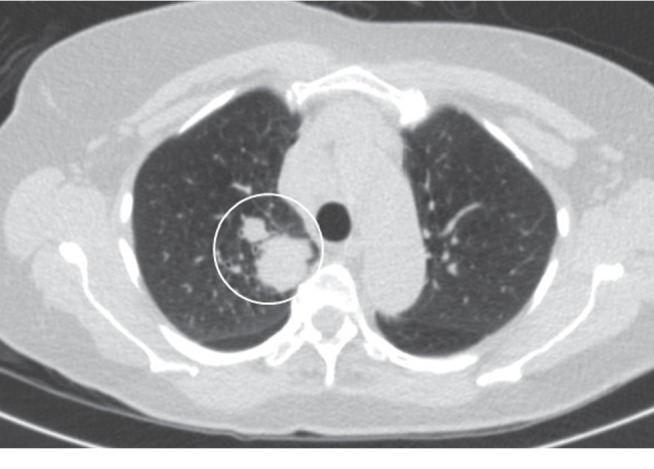
CT scan of the chest showing a 4.2 cm lobulated pleural-based mass in the right upper lobe with enlarged hilar lymph nodes suggestive of nodal metastasis.

**Figure 2:**
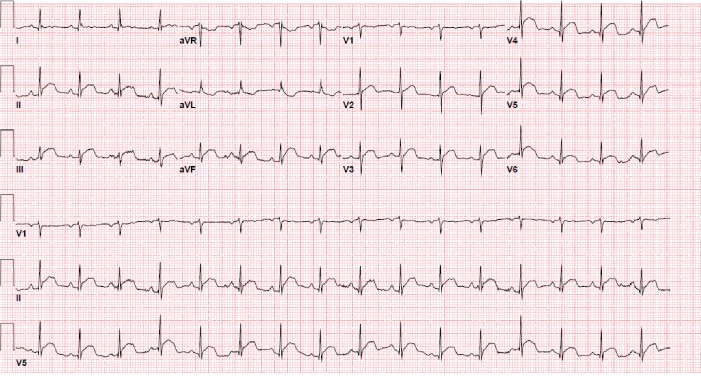
Twelve-lead electrocardiogram (EKG) performed on admission with diffuse ST-segment elevations in leads V 3–6, II, III, and aVF. Vent rate 98 bpm, PR 146 ms, QRS 76 ms, QT 314 ms, and QTc 400 ms. Ca = 19.4 mg/dl and corrected Ca for albumin = 20.4 mg/dl.

**Figure 3:**
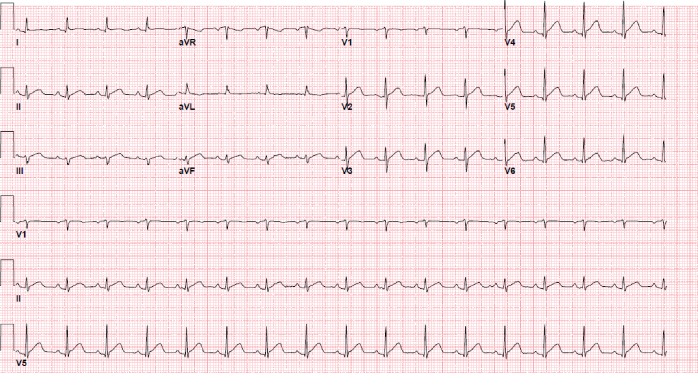
Twelve-lead EKG performed 24 hours following admission showing less marked ST-segment elevation. Vent rate 98 bpm, PR interval 150 ms, QRS duration 76 ms, QT 328 ms, and QTc 418 ms. Ca = 14.2 mg/dl and corrected Ca for albumin = 15.4 mg/dl.

**Figure 4:**
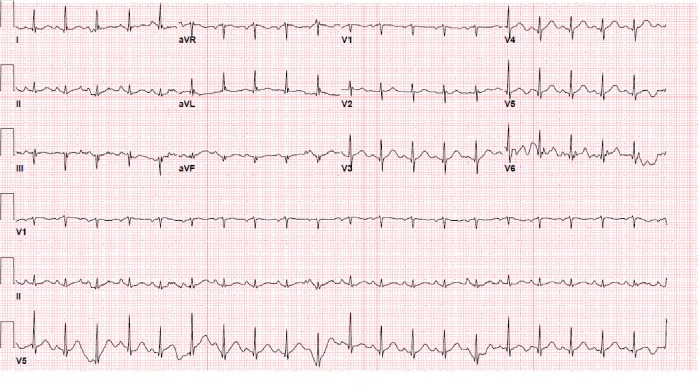
Twelve-lead EKG performed 48 hours following admission showing resolution of ST-segment elevation. Vent rate 124 bpm, PR interval 152 ms, QRS duration 74 ms, QT 310 ms, and QTc 445 ms. Ca = 11.2 mg/dl and corrected Ca for albumin = 12.4 mg/dl.

An 81-year-old female presented to the emergency room (ER) with complaints of profound fatigue, weakness, anorexia, and drowsiness. She was recently diagnosed with stage IV squamous cell cancer (SCC) of the lung (Figure 1). She also had a history of hypertension, chronic obstructive lung disease, and heavy tobacco use; however, she had no prior history of myocardial infarction or cardiac disease. Two weeks prior to admission, she had received her first session of chemotherapy. Afterward, her symptoms of fatigue and weakness progressively worsened. She also reported a vague right-sided chest pain associated with shortness of breath along with tingling sensation along the right arm. Upon arrival to the ER, she was lethargic and had blood pressure (BP) 92/55 mmHg, heart rate (HR) 101/min, and respiratory rate (RR) 24/min on room air. On physical examination, she was dehydrated and malnourished. She had normal S1 and S2 with no murmurs and no chest wall tenderness on cardiac exam. Pulmonary exam was significant for decreased breath sounds on the right side of the chest with dullness on percussion. There was also an enlarged right-sided supraclavicular lymph node. Initial laboratory studies were significant for elevated serum calcium. The uncorrected calcium was 19.6 mg/dl, serum albumin was 3 mg/dl, corrected calcium for albumin was 20.4 mg/dl (normal range 8.4–10.2 mg/dL) using the formula (corrected Ca = Ca^+^ + 0.8 ∗ (4-albumin)), and elevated PTHrP (PTH-related peptide) was 135 pg/ml. Other notable laboratory results included elevated blood urea nitrogen (BUN) 33 mg/dl, creatinine 1.33 mg/dl, elevated white blood cell (WBC) 18.2, and hemoglobin (Hb) 12.4 mg/dl. Troponin I level was normal (0.003 ng/ml). EKG revealed diffuse ST-segment elevation in leads V3–V6, I, II, III, and aVF (Figure 2). The QRS duration was 76 ms, QT interval was 314 ms, and corrected QT interval was 400 ms. Chest X-ray demonstrated a large right pleural effusion. She was treated with intravenous normal saline, calcitonin, pamidronate, and decadron. By the following day, the corrected calcium level had decreased to 15.4 mg/dl, and a repeat EKG (Figure 3) showed partial resolution of the less ST-segment elevation. The QT interval also increased from 400 to 418 ms. The EKG normalized by day 3 of treatment (Figure 4), while the calcium level reached its nadir at 11.2 mg/dL after a week of treatment.

## 3. Discussion

Hypercalcemia is a relatively frequent medical condition. It is commonly seen in the setting of malignancy where it is associated with cancer of the lung, breast, and kidneys and in multiple myeloma. In general, hypercalcemia is seen in up to 10–20% of all adults with cancer. Other etiologies for acquired hypercalcemia include hyperparathyroidism, immobilization, vitamin D intoxication, milk-alkali syndrome, sarcoidosis, and other granulomatous disorders [2]. Our patient had advanced metastatic squamous cell lung cancer in the absence of lytic bone lesions suggesting a paraneoplastic etiology for the hypercalcemia. Other contributing factors included dehydration and immobilization.

**Figure 5:**
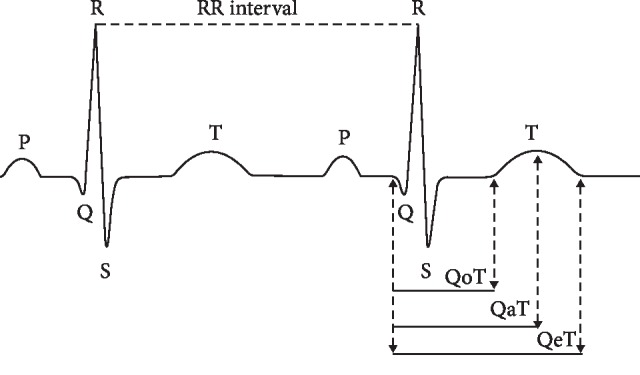
Schematic presentation of measurement of QT intervals. Corrected QT interval is calculated by dividing the QT interval by the square root of the RR interval (Bazett's formula).

Hypercalcemia is associated with distinct changes on the EKG. Most changes are transient and resolve with the correction of hypercalcemia. Classic EKG findings include a short QT interval which can be measured by calculating the distance between the beginning of the QRS complex to either the origin (QoT), the apex (QaT), or the end (QeT) of the T wave (Figure 5). The cut points for QTc below which the risk for mortality increases is 400 ms [3]. Short QoT or QaT is more specific for hypercalcemia than overall QTc intervals [4]. In our patient, the QTc interval was shortened and measured 400 ms. While short QT interval is commonly seen in hypercalcemia, other causes of acquired short QT interval include hyperkalemia or in association with medications such as digitalis which were excluded.

In general, hypercalcemia causes alteration in ionic equilibrium which changes myocardial cell membrane potentials resulting in increased myocardial contractility. The striking EKG finding seen in our patient was the presence of diffuse ST-segment elevation mimicking acute coronary syndrome. While such a finding was previously reported in the literature, mostly in case reports, in this case, we were able to trend and correlate the EKG findings from initial diagnosis till resolution of ST changes by measuring calcium levels and repeating EKGs at serial intervals [4–7]. While this patient's EKG demonstrated diffuse elevations, it also demonstrated concomitant short QT interval with a notch-shaped T wave trait more commonly associated with hypercalcemia. In light of the absence of reciprocal EKG changes, normal troponin I, and the absence of typical anginal symptoms, no further cardiac evaluation was performed. While echocardiogram was not performed in our patients, it can be helpful to improve the diagnostic specificity in selected cases. Different echocardiographic findings would suggest different disease entities. For instance, the presence of regional wall motion abnormality suggests myocardial ischemia, the presence of right ventricular (RV) strain suggests an underlying pulmonary embolism, and the presence of dissection flap in ascending aorta can be seen in aortic dissection.

EKG of a hypercalcemic patient may also demonstrate prolongation of the PR and QRS intervals, increased amplitude of the QRS complex, Osborn (J) waves, J point elevation (including early repolarization and Brugada-type EKG), biphasic inverted or notched T waves, and prominent U waves [8, 9]. In a large case series performed by Littman et al., a total of 16 cases of severe hypercalcemia with concomitant ST-segment elevation on EKGs mimicking acute coronary syndrome were described. The most common etiology for hypercalcemia in this population was malignancy, followed by hyperparathyroidism. The majority of patients were males (75%), the mean serum calcium level was 14.3 ± 2.9 mg/dL, and ST-segment elevations were most commonly seen in anterior chest leads and showed a characteristic “scooped” appearance and were not followed by a distinct T wave [10]. Our patient had a strikingly elevated serum calcium level on admission that was far higher than the average calcium level reported by Littman et al. Moreover, the serial follow-up of EKG changes with hypercalcemia was corrected as hypercalcemia resolved.

Prior investigation into the mechanism of hypercalcemia-induced EKG changes by Kazama et al. used an animal model to study the change in action potential in the setting of elevated calcium. A highly concentrated calcium solution was added to the surface of the heart, and dual recording of both myocardial cell action potential and the EKG was performed. Two minutes following exposure of the cardiac tissue to the highly concentrated calcium solution, action potential recording showed that the slope of phase 2 became steeper and the duration became shorter with unchanged duration of phase 3. This change was accompanied by a hump at the end of the widened QRS complex in the EKG (Osborn or J wave). Thirty minutes later, the slope of phase 3 became steeper and duration became shorter, which correlated with the absence of Osborn waves on the EKG and markedly shortened QT intervals [11].

The mechanism of hypercalcemia-induced ST-segment elevation is not fully understood. It is hypothesized that hypercalcemia-induced shortening of the QT interval might come at the expense of the ST-segment, causing the T waves to be pulled to the end of the QRS complex causing the appearance of ST-segment elevation. This hypothesis is further supported by the scooped appearance of the segment with absence of additional deflections which would correlate with T wave following QRS complexes [10]. Other possibilities include development of a biphasic or flattened T wave that mimics an ST-segment elevation. In addition, the altered equilibrium of ionic flow across the potassium and calcium channels can also lead to such ST-segment changes [5]. Other differentials to be considered for evaluation of ST-segment elevation in absence of ACS include early repolarization, pericarditis, hypertrophic cardiomyopathy, increased intra cranial pressure, and Brugada syndrome (Table 1).

In general, while J point elevation including early repolarization and Brugada-type EKG are associated with higher risk for ventricular tachyarrhythmias, such risk is not common in the setting of hypercalcemia. In a study conducted by Sonoda et al., 89 patients with hypercalcemia were analyzed. Elevated J point was seen in 30% of the total; however, no fatal arrhythmia was observed during the study period [9]. Conversely, in few case reports, hypercalcemia and a Brugada-type pattern had resulted in ventricular fibrillation [14]. Hypercalcemia-induced ventricular arrhythmia is a rare phenomenon and is particularly more common in association with hyperparathyroidism. Parathormone has an independent positive inotropic as well as chronotropic effect on cardiac myocytes which, in the presence of hypercalcemia, results in decreased ventricular conduction velocity and shortened refractory period causing higher chance for re-entry and development of ventricular fibrillation [8, 15].

The management of hypercalcemia depends on the severity and presence of symptoms. For acutely symptomatic patients and those with moderate to severe hypercalcemia (calcium >12 mg/dl), prompt management and hospital admission is required. The goal of treatment is to promote renal calcium excretion with intravenous (IV) fluids and prevent further bone resorption. Loop diuretics are not recommended unless kidney failure or heart failure is present, in which case volume expansion should precede the administration of loop diuretics to avoid hypotension and further kidney injury. Calcitonin is useful in hypercalcemia refractory to saline diuresis. It is used to acutely lower the calcium levels (effective within 4–6 hours) with tachyphylaxis occurring after approximately 3 days, likely due to downregulation of calcitonin receptors on osteoclasts. Glucocorticoids are used in the setting of hypercalcemia mediated by vitamin D–secreting tumors or lymphomas. Bisphosphonates are first-line therapy and also the mainstay for long-term therapy (effective within 2 to 4 days). Bisphosphonate therapy requires adequate kidney function, and caution should be exercised with these agents on the setting of kidney dysfunction. In patients resistant to or intolerant of bisphosphonate therapy, off-label use of denosumab, which also reduces osteoclast-mediated bone resorption, can be recommended. For patients who present with serum calcium levels greater than 18 mg/dL (4.5 mmol/L), with neurologic symptoms or compromised kidney function, especially oliguric patients, hemodialysis is an appropriate choice to quickly reduce calcium levels [16, 17].

## 4. Conclusion

In the absence of clinical suspicion of acute coronary syndrome, the interpretation of the EKG should be carefully integrated with consideration of the overall clinical picture. Hypercalcemia needs to be considered in the differential diagnosis of ST-segment elevation in absence of acute coronary syndrome. The presence of short QT interval further supports this diagnosis. Awareness of electrophysiological changes related to hypercalcemia is important for early recognition and appropriate treatment.

## Figures and Tables

**Table 1:** Differential diagnosis for ST-segment elevation.

Condition	ST-segment morphology	Other EKG findings
ACS	Concave, convex, or obliquely straight morphology in contiguous leads	Presence of reciprocal changes

Benign early repolarization	Concave morphology, especially in V2–V5	Possible to have slurring or notching at J point

Acute pericarditis	Diffuse, with upward concavity.	PR-segment depression except for aVR where it is elevated

Hypertrophic cardiomyopathy [12]	ST elevation with voltage criteria for LVH	Repolarization changes and giant (>10 mm) inverted T waves in the anterolateral leads

Brugada syndrome	Coved ST-segment elevation >2 mm in >1 of V1–V3	ST elevation is followed by a negative T wave

LV aneurysm [13]	Persistent ST-segment elevation in contiguous leads	Pathologic Q waves
